# Identification of the Tyrosine-Protein Phosphatase Non-Receptor Type 2 as a Rheumatoid Arthritis Susceptibility Locus in Europeans

**DOI:** 10.1371/journal.pone.0066456

**Published:** 2013-06-20

**Authors:** Joanna E. Cobb, Darren Plant, Edward Flynn, Meriem Tadjeddine, Philippe Dieudé, François Cornélis, Lisbeth Ärlestig, Solbritt Rantapää Dahlqvist, George Goulielmos, Dimitrios T. Boumpas, Prodromos Sidiropoulos, Sophine B. Krintel, Lykke M. Ørnbjerg, Merete L. Hetland, Lars Klareskog, Thomas Haeupl, Andrew Filer, Christopher D. Buckley, Karim Raza, Torsten Witte, Reinhold E. Schmidt, Oliver FitzGerald, Douglas Veale, Stephen Eyre, Jane Worthington

**Affiliations:** 1 Arthritis Research UK Epidemiology Unit and NIHR Manchester Musculoskeletal BRU, Central Manchester Foundation Trust, Manchester Academic Health Science Centre, The University of Manchester, Manchester, United Kingdom; 2 GenHotel-EA3886-4679, Auvergne and Evry University, Clermont-Ferrand, France; 3 Rheumatology Department and INSERM U699, Bichat Hospital, Assistance Publique – Hôpitaux de Paris and Paris 7 University, Paris, France; 4 Institution of Public Health and Clinical Medicine/Rheumatology, Umeå University, Umeå, Sweden; 5 Clinic of Rheumatology, Department of Medicine, University of Crete, Heraklion, Greece; 6 DANBIO registry, Department of Rheumatology, Glostrup Hospital, Faculty of Health Sciences, University of Copenhagen, Glostrup, Denmark; 7 Department of Medicine, Karolinska Institutet, Karolinska University Hospital, Stockholm, Sweden; 8 Department of Rheumatology and Clinical Immunology, Charité University Hospital, Berlin, Germany; 9 Rheumatology Research Group, The University of Birmingham, Birmingham, United Kingdom; 10 Sandwell and West Birmingham Hospitals NHS Trust, Birmingham, United Kingdom; 11 Department of Immunology and Rheumatology, Hannover Medical School, Hannover, Germany; 12 Department of Rheumatology, St Vincent's University Hospital, UCD School of Medicine and Medical Sciences and Conway Institute of Biomolecular and Biomedical Research, University College Dublin, Dublin, Ireland; Kunming Institute of Zoology, Chinese Academy of Sciences, China

## Abstract

**Objectives:**

Genome-wide association studies have facilitated the identification of over 30 susceptibility loci for rheumatoid arthritis (RA). However, evidence for a number of potential susceptibility genes have not so far reached genome-wide significance in studies of Caucasian RA.

**Methods:**

A cohort of 4286 RA patients from across Europe and 5642 population matched controls were genotyped for 25 SNPs, then combined in a meta-analysis with previously published data.

**Results:**

Significant evidence of association was detected for nine SNPs within the European samples. When meta-analysed with previously published data, 21 SNPs were associated with RA susceptibility. Although SNPs in the *PTPN2* gene were previously reported to be associated with RA in both Japanese and European populations, we show genome-wide evidence for a different SNP within this gene associated with RA susceptibility in an independent European population (rs7234029, *P* = 4.4×10^−9^).

**Conclusions:**

This study provides further genome-wide evidence for the association of the *PTPN2* locus (encoding the T cell protein tyrosine phosphastase) with Caucasian RA susceptibility. This finding adds to the growing evidence for *PTPN2* being a pan-autoimmune susceptibility gene.

## Introduction

A European Union funded project, the AutoCure consortium (www.autocure.org) involves collaboration between 26 European multinational partners in the search for improved understanding of inflammatory rheumatic diseases. To co-ordinate the collection of DNA for analysis, the Genetic Repository for AutoCure (GRACE) was established. GRACE incorporates rheumatoid arthritis (RA) patient samples from Denmark, France, Germany, Greece, Ireland, Sweden and UK, and controls from all populations except Denmark. A recent publication used the GRACE cohort to investigate RA loci [1]. Since then, additional samples have been added to GRACE, and evidence has emerged from other well powered RA cohorts for association to additional SNPs, although not all of these have achieved genome wide significance (*P*<5×10^−8^). Therefore, this study aimed firstly to continue characterising the GRACE cohort for confirmed RA susceptibility loci, and secondly to genotype SNPs for provisional RA loci in GRACE and perform a meta-analysis using previously published data to investigate whether any can be confirmed at genome wide significance, therefore providing further evidence for the role of these variants in the susceptibility of RA.

## Methods

### Cohorts

All samples included in this analysis were of European ancestry, with RA as defined by the 1987 American College of Rheumatology classification criteria for genetic studies [2]. Demographics were collected where available. A total of 4286 RA subjects were included from nine centres across Europe. To minimise potential problems with population stratification, a total of 5642 controls were obtained from the same populations as each GRACE cohort, with the exception of the Danish samples for which controls were unavailable. To account for this the genotype counts between the Swedish and Danish cases were compared for each SNP using a χ^2^ test (or Fisher’s exact test when genotype counts were less than 10) and found to not significantly differ (Bonferroni corrected *P*<0.002, data not presented) as seen previously for these cohorts [1]. The Scandinavian cases were therefore combined prior to analysis with Swedish controls. Similarly, the UK cases from Manchester and Birmingham were found not to differ significantly, were combined and compared to controls available via the Wellcome Trust Case Control Consortium 2 (WTCCC2). The Dublin control data was obtained from a previous study [3]. Ethical approval was obtained via written informed consent and in accordance with the Declaration of Helsinki from the following bodies: North West Multicentre Research Ethics Committee (Manchester, UK), Sandwell and West Birmingham Research Ethics Committee and Solihull Research Ethics Committee (Birmingham, UK), Regional Ethics Committee at the University Hospital of Umeå (Umeå, Sweden), Ethical Committee of the Capital Region (Copenhagen, Denmark), St. Vincent's University Hospital Ethics Committee (Dublin, UK)**,** Research Ethics Committee of the Faculty of Medicine, University of Crete (Crete, Greece), Local Ethical Committee of the Hannover Medical School (Hannover, Germany), Charité Local Ethical Committee (Berlin, Germany), Ethics Committee of Hôpital Kremlin-Bicêtre (Paris, France).

### Genotyping

Twenty-five markers were selected to further characterise the GRACE cohort and validate unconfirmed RA SNPs based on recent findings [4]–[6]. Group 1 SNPs have previously been shown to have genome-wide association with RA susceptibility (*P*<5×10^−8^) in a meta-analysis published by Stahl and colleagues [6], group 2 SNPs have prior evidence at genome-wide significance level in other RA studies, and group 3 SNPs are potential RA loci identified in the Stahl meta-analysis which have not yet been confirmed at genome-wide significance (see Tables 1 and 2). Sequenom MassArray multiplex assays were designed and genotyping performed using iPLEX chemistry as per the manufacturer’s instructions (Sequenom Inc., USA). Only SNPs with genotyping success rate >95% and samples with success rate >90% were included in the analysis.

**Table 1:** Results from initial (GRACE only) meta-analysis.

SNP	Gene	Case/Control		MAF Case		MAF Control		Het *P*	OR (95% CI)	?2*P*	
**Group 1: SNPs with previous evidence of genome-wide association with RA susceptibility from Stahl meta-analysis**											
rs10488631	*IRF5*	3193/5018		0.12		0.12		0.748	1.06 (0.95,1.17)	0.297	
rs13315591	*PXK-FAM107A*	3194/5020		0.07		0.07		0.798	1.03 (0.91,1.18)	0.612	
rs26232	*C5orf30/GIN1*	3038/5014		0.3		0.33		0.98	0.88 (0.82,0.94)	4.68 ×10^−4^	
rs3093023	*CCR6*	3037/5020		0.47		0.44		0.321	1.09 (1.02,1.17)	0.012	
rs6859219	*ANKRD55*	3026/4950		0.18		0.21		0.792	0.87 (0.8,0.95)	0.002	
rs706778	*IL2RA*	3007/4998		0.43		0.41		0.157	1.04 (0.97,1.11)	0.301	
rs874040	*RBPJ*	3195/5003		0.32		0.29		0.235	1.14 (1.07,1.23)	2.18 ×10^−4^	
rs951005	*CCL21*	2857/4857		0.16		0.17		0.72	0.92 (0.84,1.01)	0.069	
**Group 2: SNPs with previous evidence of genome-wide association with RA susceptibility from other studies**											
rs11586238^†^	*CD2,CD58*	3195/5015		0.25		0.23		0.292	1.12 (1.03,1.21)	0.005	
rs13031237^†^	*REL*	3191/4952		0.37		0.36		0.147	1.06 (0.99,1.13)	0.125	
rs1980422^†^	*CD28*	3093/4960		0.25		0.23		0.663	1.15 (1.07,1.25)	2.98×10^−4^	
rs2736340^†^	*BLK*	3194/5021		0.26		0.24		0.655	1.14 (1.05,1.22)	0.001	
rs548234^†^	*PRDM1*	3192/5010		0.33		0.33		0.062	0.87 (0.8,0.95)	0.205	
**Group 3: SNPs not previously associated with RA susceptibility to a genome-wide significance level**											
rs10865035	*AFF3*	3189/2286		0.48		0.47		0.539	1.02 (0.94,1.11)		0.61
rs11203203	*UBASH3A*		3176/5009		0.38		0.37	0.196	1.00 (0.93,1.07)		0.911
rs11594656	*IL2RA*		3195/5014		0.24		0.25	0.534	0.98 (0.91,1.06)		0.579
rs394581	*TAGAP*		3190/4942		0.28		0.3	0.531	0.91 (0.84,0.98)		0.008
rs42041	*CDK6*		3186/5015		0.25		0.24	0.277	1.06 (0.98,1.14)		0.148
rs4535211	*PLCL2*		3195/5107		0.46		0.48	0.102	0.96 (0.9,1.03)		0.237
rs540386	*TRAF6*		2787/4600		0.13		0.14	0.071	1.05 (0.98,1.12)		0.207
rs7234029	*PTPN2*		3189/5014		0.18		0.16	0.092	1.15 (1.05,1.26)		0.002
rs7543174	*UBE2Q1*		3194/4951		0.18		0.18	0.056	1.03 (0.95,1.12)		0.508

SNPs allocated to group 1, 2 or 3 based on previously published evidence. The SNPs^†^ in group 2 do not reach genome-wide significance in Stahl et al used in meta-analysis [6] (Table 2), but have in prior publications [4], [5]. Het *P* = meta-analysis heterogeneity χ^2^ *P* value, OR = odds ratio calculated using fixed or random* effects meta-analysis.

**Table 2:** SNP results from previously published associations of RA susceptibility and the meta-analysis results now GRACE (AutoCure) data has been added.

SNP	Gene	Previously published evidence for SNPs prior to GRACE meta-analysis				Meta-analysis of GRACE plus previously published evidence						
		MAFCase	MAFControl	OR (95% CI)	?^2^ *P*	Case/Control	MAFCase	MAFControl	Het *P*	OR (95% CI)	?^2^ *P*	Power(%)ß
**Group 1: SNPs with previous evidence of genome-wide association with RA susceptibility from Stahl meta-analysis**												
rs10488631	*IRF5*	0.13	0.1	1.21 (1.14,1.28)	4.2×10^−11^	7031/10245	0.13	0.1	0.008	1.08* (0.97, 1.21)	1.10×10^−15^	57
rs13315591	*PXK-FAM107A*	0.09	0.08	1.20 (1.12,1.28)	4.6×10^−8^	9639/13084	0.08	0.08	0.699	1.11 (1.04, 1.19)	0.002	86
rs26232	*C5orf30/GIN1*	0.3	0.32	0.90 (0.87,0.94)	4.1×10^−8^	9450/13037	0.3	0.32	0.963	0.9 (0.87, 0.94)	1.51×10^−7^	100
rs3093023	*CCR6*	0.46	0.43	1.12 (1.08,1.16)	1.5×10^−11^	9436/13052	0.46	0.43	0.363	1.12 (1.08, 1.16)	1.08×10^−9^	100
rs6859219	*ANKRD55*	0.19	0.21	0.81 (0.77,0.86)	9.6×10^−12^	5785/8965	0.19	0.21	0.876	0.88 (0.84, 0.92)	2.98×10^−8^	100
rs706778	*IL2RA*	0.43	0.4	1.12 (1.09,1.16)	1.4×10^−11^	9428/13012	0.43	0.4	0.053	1.04* (0.99, 1.08)	8.32×10^−8^	52
rs874040	*RBPJ*	0.34	0.3	1.16 (1.12,1.20)	1.0×10^−16^	9592/13012	0.33	0.3	0.226	1.19 (1.14, 1.23)	2.18×10^−18^	100
rs951005	*CCL21*	0.13	0.15	0.86 (0.82,0.90)	3.9×10^−10^	8981/12419	0.14	0.15	0.573	0.87 (0.82, 0.91)	5.53×10^−8^	100
**Group 2: SNPs with previous evidence of genome-wide association with RA susceptibility from other studies**												
rs11586238^†^	*CD2,CD58*	0.26	0.23	1.13 (1.07,1.19)	1.0×10^−5^	8732/25176	0.25	0.23	0.35	1.14 (1.1, 1.19)	2.83×10^−10^	100
rs13031237^†^	*REL*	0.39	0.36	1.13 (1.07,1.18)	7.9×10^−7^	8728/25113	0.38	0.36	0.077	1.11 (1.07, 1.15)	1.23×10^−8^	100
rs1980422^†^	*CD28*	0.26	0.24	1.12 (1.06,1.18)	5.2×10^−5^	8630/25120	0.26	0.24	0.697	1.12 (1.08, 1.17)	2.40×10^−8^	100
rs2736340^†^	*BLK*	0.27	0.25	1.12 (1.07,1.18)	1.5×10^−5^	8733/25181	0.27	0.25	0.732	1.12 (1.07,1.16)	7.90×10^−8^	100
rs548234^†^	*PRDM1*	0.34	0.33	1.10 (1.05,1.16)	9.7×10^−5^	8724/25171	0.34	0.33	0.1	1.05 (1.01, 1.09)	0.019	75
**Group 3: SNPs not previously associated with RA susceptibility to a genome-wide significance level**												
rs10865035	*AFF3*	0.5	0.47	1.12 (1.07,1.17)	2.0×10^−6^	8725/22440	0.49	0.47	0.217	1.11 (1.07, 1.15)	1.83×10^−7^	100
rs11203203	*UBASH3A*	0.38	0.37	1.09 (1.05,1.13)	3.8×10^−6^	7269/10093	0.38	0.37	0.194	1.03 (0.99, 1.07)	0.119	26
rs11594656	*IL2RA*	0.24	0.25	0.92 (0.89,0.96)	1.0×10^−4^	8856/13274	0.24	0.25	0.592	0.96 (0.92, 1)	0.035	44
rs394581α	*TAGAP*	0.27	0.3	0.91 (0.87,0.96)	0.001	8727/25102	0.27	0.3	0.5	0.88 (0.84, 0.91)	4.44×10^−11^	100
rs42041	*CDK6*	0.28	0.26	1.11 (1.05,1.17)	1.0×10^−4^	8722/25180	0.27	0.26	0.291	1.09 (1.05, 1.14)	1.47×10^−5^	100
rs4535211	*PLCL2*	0.46	0.49	0.92 (0.88,0.97)	0.001	8728/25256	0.46	0.49	0.039	0.98* (0.94, 1.02)	9.92×10^−8^	21
rs540386	*TRAF6*	0.13	0.14	0.88 (0.83,0.94)	3.0×10^−4^	8326/24766	0.13	0.14	0.114	0.92 (0.88, 0.97)	0.003	89
rs7234029	*PTPN2*	0.18	0.16	1.13 (1.06,1.20)	1.0×10^−4^	8724/25175	0.18	0.16	0.144	1.15 (1.1, 1.21)	4.44×10^−9^	100
rs7543174	*UBE2Q1*	0.19	0.18	1.10 (1.06,1.15)	1.2×10^−5^	9624/13002	0.19	0.18	0.076	1.06 (1.01, 1.11)	0.018	66

SNPs allocated to group 1, 2 or 3 based on previously published evidence. The SNPs^†^ in group 2 do not reach genome-wide significance in Stahl et al used in this meta-analysis [6], but have in prior publications [4], [5].

α Additionally, the TAGAP locus has previously been associated with RA at a genome-wide level, however not for the SNP investigated here [19]. Het *P* = meta-analysis heterogeneity χ^2^ *P* value, OR = odds ratio calculated using fixed or random* effects meta-analysis.

ß Power calculation based on these results assuming 1% disease prevalence in the population.

### Statistical Analysis

Allele counts and genotype frequencies were calculated for each population, analysed using an additive genetic model, and combined in a meta-analysis of all GRACE cohorts. Published data was used to perform a second meta-analysis with the GRACE data [6], Stahl’s study selected as it is the largest and most comprehensive meta-analysis of RA to date. A fixed effect model (Mantel-Haenszel method) was used to perform the meta-analysis, with cohorts weighted based on the amount of information they contain. Where evidence for between cohort heterogeneity (I^2^) was observed (*P*<0.05) a random effects model (DerSimonian and Laird method) was used, as indicated in the results.

Power calculations were performed at the 5% significance level using previously published effect sizes and for the results of the second meta-analysis using the software package Quanto v1.2 (http://hydra.usc.edu/gxe). All analysis was undertaken using the statistical software packages Stata v10 (StataCorp 2009) and Plink v1.07 [7].

## Results and Discussion

### Cohort Characteristics and Quality Control

The AutoCure consortium facilitated the establishment of GRACE, a large collection of pan-European DNA samples. A total of 3195 RA subjects (Manchester 820, Birmingham 95, Umeå 665, Copenhagen 286, Paris 307, Crete 402, Hannover 199, Berlin 265, Dublin 156) and 5378 controls passed QC with genotyping success rate >90%. The SNPs selected for this study were based on a recent publication that identified seven novel RA susceptibility loci at genome-wide significance and additional markers with suggestive evidence for association [6]. In the GRACE analysis, one SNP (rs540386 in *TRAF6*) in the Cretan cohort showed significant deviations from Hardy-Weinberg equilibrium in controls and was therefore excluded from the analysis for these samples. There was no data available for rs10865035 in *AFF3* (nor an available proxy) in the UK control data from the WTCCC2, therefore this SNP was excluded in the UK samples. Two SNPs in the UK control data from the WTCCC2 were unavailable, however perfect proxies (r^2^ = 1) were used for rs10488631 (proxy rs12531711) and rs26232 (proxy rs2288786). The RA cases from Dublin were added to the GRACE collection at a later stage and were therefore genotyped separately. For these samples, an additional five SNPs failed genotyping QC (rs26232, rs3093023, rs6859219, rs706778 and rs951005) and were therefore excluded from the analysis for the Dublin samples. Three group 3 SNPs failed QC due to genotyping success of <95% (rs10919563 near *PTPRC*, rs12746613 near *FCGR2A,* and rs3184504 near *SH2B3*).

### Meta-analysis of GRACE Cohorts

An association analysis of the additive genetic model for each SNP genotyped in the GRACE cohorts was initially performed (data not presented). These results were then combined in a meta-analysis, which replicated the association of nine SNPs with RA (*P*<0.05, Table 1). The strongest associations were seen with SNPs rs26232 (*GIN1 P* = 4.68×10^−4^), rs874040 (*RBPJ P* = 2.18×10^−4^) and rs1980422 (*CD28 P* = 2.98×10^−4^).

### Overall Meta-analysis, Including Previously Published Data

The meta-analysis of previously published data with the new GRACE genotyping provided further evidence for the role of 21 SNPs in the susceptibility of RA in European populations and improved the previously published association signal for eight SNPs (Table 2) [4]–[6]. We improved the association at *RBPJ* (rs874040) and *IRF5* (rs10488631); replicate the association at *ANKRD55-IL6ST* (rs6859219), *C5orf30/GIN1* (rs26232) and *CCR6* (rs3093023); and replicate the association at *PXK-FAM107A* (rs13315591), although the evidence for this locus has reduced and thus needs to be further investigated in an independent population. Other SNPs genotyped resulted in reduced association signals. Three SNPs (rs10488631, rs4535211 and rs706778) had significant between-study heterogeneity, primarily due to the broad distribution of effect sizes in different directions between studies.

Notably, we increased the previously reported association of a SNP [1], [6] within an intron of *PTPN2* to a genome-wide significance level for the first time in a European population of RA patients (rs7234029, OR = 1.15, *P* = 4.44×10^−9^). This same variant is highly associated with juvenile idiopathic arthritis [8], [9]. More recently another SNP within *PTPN2* (rs2847297) was found to be associated with RA in a Japanese population at the genome-wide significance level (*P* = 2.2×10^−8^) [10], and a further SNP (rs62097857) in low linkage disequilibrium with rs7234029 has recently been implicated (*P* = 4.4×10^−6^) in a large Caucasian study of RA [11]. However, the SNP detailed in this publication by Eyre *et al* is in low LD with rs7234029 tested here, and as this region fails to reach the significance thresholds set by Eyre *et al* there is little detail of the *PTPN2* results within the article or supplementary files published, inhibiting more detailed comparisons. This highlights the need for further investigation of the role of *PTPN2* in RA pathogenesis in both European and Asian populations. *PTPN2* is a non-receptor tyrosine-protein phosphatase similar to *PTPN22*. It encodes the T cell protein tyrosine phosphastase, which is known to act as a negative regulator of the JAK/STAT signalling pathways downstream of cytokines such as IL2, and thus may play an important role in T cell activation [12]. The results presented here add to the mounting evidence in RA [10], [11], along with studies in Crohn’s disease [13], type 1 diabetes [14], and juvenile idiopathic arthritis [9], to provide evidence for *PTPN2* being a pan-autoimmune susceptibility gene.

Interestingly evidence for rs4535211, within the negative regulator of B cell receptor signalling (*PLCL2*) gene, was increased from *P* = 0.001 in the Stahl meta-analysis to *P* = 9.92×10^−8^, approaching genome-wide significance levels. This SNP has been highlighted in a recent psoriatic arthritis (PsA) study as being associated with early age of onset disease, although the allele that confers protection for RA here is a risk allele for PsA [15].

One limitation to this work is that the collection of samples utilised contains both seropositive and seronegative RA patients. There is continued debate surrounding the genetic differences between RA patients with different serology [16], [17], and therefore the GRACE cohort may only have the power to detect associations with SNPs involved in both types of RA. A further limitation is the potential for population stratification and bias by combining Danish and Swedish cases in the analysis with only Swedish controls. Although the relatively small numbers of Scandinavian RA cases available reduces our power to detect differences at these SNPs, no evidence for bias within these cohorts was observed (lack of significant differences in genotype frequencies between the cases), in concordance with previous studies in these samples [1]. Unfortunately the GRACE cohort only has limited genotyping data available and therefore insufficient unlinked markers for population stratification methods such as genomic control to test this more thoroughly.

This study utilises the large GRACE resource of up to 10,000 European RA cases and controls to provide further evidence for the role of 21 SNP markers in the susceptibility to RA in European populations. This has enabled detection of genome-wide evidence for the association of a SNP within an intron of *PTPN2* which, along with recent investigations of different SNPs at this locus, highlights the need for more comprehensive investigation at this locus in large RA cohorts from multiple ethnicities. Further fine-mapping of these regions will be required to identify the causal variants involved in RA susceptibility. Such work is now possible within the Immunochip, a custom Illumina Infinium genotyping chip designed to fine-map genomic loci involved in multiple autoimmune diseases [18].
